# Determinants of Postnatal Care Utilization Among Mothers in Degahbour District, Somali Region, Ethiopia: A Community‐Based Cross‐Sectional Study

**DOI:** 10.1155/jp/4926845

**Published:** 2026-07-09

**Authors:** Ayderus Abdirahman, Arega Abebe Lonsako, Merga Deresa, Haymanot Mezmur

**Affiliations:** ^1^ Health Office, Degehabur, Somali Region, Ethiopia; ^2^ College of Medicine and Health Sciences, Arba Minch University, Arba Minch, Ethiopia, amu.edu.et; ^3^ College of Health and Medical Sciences, Haramaya University, Harar, Ethiopia, haramaya.edu.et

**Keywords:** determinants, Ethiopia, mothers, postnatal care utilization

## Abstract

**Background:**

Postnatal care (PNC) refers to the care provided to women and their newborns during the first 42 days after birth. Insufficient care during this critical period can lead to severe health outcomes, including disability or death. Despite its crucial role in enhancing maternal and neonatal health, PNC services remain underutilized in Ethiopia, with limited evidence available regarding their use in the Somali Region. Therefore, this study is aimed at evaluating the magnitude of PNC service utilization and identify the factors influencing it among women who gave birth in the past 6 months in Degahbour District, Somali Region, Ethiopia.

**Methods:**

A community‐based cross‐sectional study was conducted among 403 mothers in the Degahbour District. Participants were selected using a systematic random sampling technique. Data were collected using a pretested, structured questionnaire, entered into EpiData Version 4.6, and analyzed using the Statistical Package for the Social Sciences (SPSS) Version 26. A logistic regression model was applied, and statistical significance was determined at p < 0.05.

**Result:**

The magnitude of PNC service utilization was 40.2% (95% CI: 35.0, 44.9). Significant factors associated with PNC utilization included complications experienced during delivery (AOR: 3.93; 95% CI: [2.33, 6.64]), higher educational attainment (secondary level or above) (AOR: 3.45; 95% CI: [1.61, 7.37]), attending antenatal care (ANC) during the last pregnancy (AOR: 3.36; 95% CI: [2.04, 5.54]), maternal autonomy in healthcare decision‐making (AOR: 2.23; 95% CI: [1.39, 3.57]), place of delivery (AOR: 1.97; 95% CI: [1.20, 3.24]), and awareness of PNC (AOR: 1.83; 95% CI: [1.14, 2.92]).

**Conclusion:**

The utilization of PNC services was found to be low. Enhancing awareness about PNC, promoting ANC visits, and encouraging institutional deliveries are critical steps to improve PNC service utilization.

## 1. Introduction

According to the World Health Organization (WHO), postnatal care (PNC) is defined as the care provided to a woman and her newborn child within hours of the placenta′s birth and for the first 42 days of life [1]. WHO recommends healthy women and neonates should receive PNC in the institution for at least 24 h following an uncomplicated birth in a healthcare facility. If the baby is born at home, the first postpartum contact should happen as soon as possible, ideally within 24 h. All mothers and newborns should have at least three more postpartum visits: on Day 3, between Days 7 and 14 after delivery, and 6 weeks after delivery [2], whereas the Federal Ministry of Health in Ethiopia recommends four PNC care visits spaced out over the course of 6 h, a day, a week, and 6 weeks [3].

In 2017, an estimated 295,000 maternal deaths occurred around the world; 99% of these deaths occurred in developing regions. Approximately 810 women die each day from pregnancy‐ and childbirth‐related problems, and [4] around 57% of maternal deaths worldwide take place in the postpartum period [5, 6]. The majority of maternal and neonatal deaths occur within the first 42 days; over half of postpartum maternal deaths occur during the first 24 h, and 66% occur within the first week [7]. In low‐income countries, almost 40% of women experience complications after delivery, and an estimated 15% develop potentially life‐threatening problems [8].

Even though using PNC is one of the most essential indicators for decreasing maternal and infant mortality, only 63% of mothers and 48% of babies worldwide use PNC services within the advised timeframe, and less than 13% of mothers in sub‐Saharan Africa countries receive PNC within 2 days of delivery [9]. In 2019, only 34% of Ethiopian women received postpartum care; this figure ranges greatly from 10% in the Somali area to 74% in Addis Ababa [10].

It was indicated that PNC utilization is influenced by place of delivery, experiencing complications during and after labor, and advice during antenatal care (ANC) visits [11] residence, maternal age, maternal education, maternal occupation, media exposure [9], autonomy in decision‐making, husband′s occupation, wealth status [4], and parity [12].

Despite its significant role in improving maternal and neonatal health, the PNC services are underutilized in Ethiopia, and there is a paucity of evidence in Somali Region. Thus, the aim of this study was to assess the magnitude of PNC service utilization and associated factors among women who gave birth in the last 6 months in Degahbour District, Somali Region, Ethiopia.

### 1.1. Conceptual Framework

**Figure 1:**
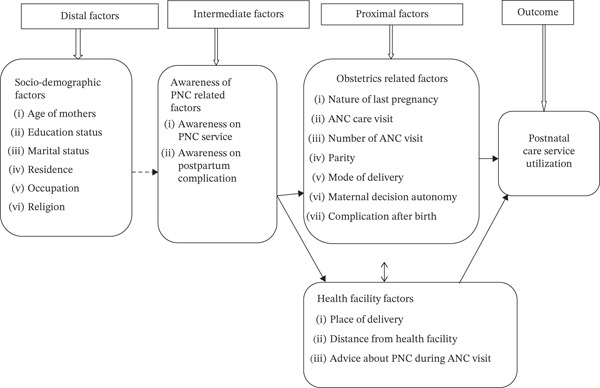
Conceptual framework shows the determinants of postnatal care service utilization.

The conceptual framework for this study was adapted from relevant literature. It includes sociodemographic, obstetric, and health service related factors as independent variables influencing PNC service utilization. These variables include parity, ANC attendance, place and mode of delivery, delivery complications, awareness of PNC and danger signs, maternal decision‐making autonomy, child illness during the postnatal period, and access to health facilities. The dependent variable was utilization of PNC services after childbirth. The framework guided the analysis of associations between the independent variables and PNC utilization (Figure 1).

## 2. Methods and Materials

### 2.1. Study Design, Area, and Period

A community‐based cross‐sectional study was conducted in Degahbour District, Jarar Zone, Somali Region, Ethiopia from March 1 to April 1, 2023. It is the administrative center of Jarar Zone, located 170 km to the East of Jigjiga City and 780 km away from Addis Ababa Ethiopia′s capital. The district has 18 kebeles (2 urban and 16 rural). The total population of the Degahbour District is 76,533 of whom 39,350 are women. The estimated number of women of childbearing age (15–49 years) is 17,487 in 2022 [13]. The main ethnic group is Somali, and most residents are Muslim. There is one general hospital, four health centers, and 22 health posts in the district. According to the report of the Degahbour District Health Office, the coverage of ANC is 67% [14].

### 2.2. Populations

All mothers of childbearing age who had given birth within the last 6 months in Degahbour District were considered the source population. The study population comprised mothers of childbearing age who had given birth within the last 6 months and were residing in randomly selected kebeles of Degahbour District at the time of data collection.

### 2.3. Eligibility Criteria

Mothers who had given birth within the last 6 months and had been residing in Degahbour District for at least 6 months prior to the study were included. Mothers with severe mental impairments that hindered communication were excluded from the study.

### 2.4. Sample Size Determination and Sampling Techniques

The sample size was calculated by using a single population proportion formula with the following assumptions: 95% confidence level, 5% margin of error, and proportion of women using PNC services 39.1% [15], which is taken from a study done in Eastern Ethiopia. So, the final calculated sample size with a 10% nonresponse rate was 403.

Degahbour District was intentionally selected as the study site due to concerns about the health status of women in the district and the lack of previous research on maternal healthcare services in the area. Out of the 15 kebeles in the district (2 urban and 13 rural), five kebeles (1 urban and 4 rural) were selected through simple random sampling. The total number of households with mothers who had given birth in the last 6 months was obtained from each Kebele administration, and a sampling frame was prepared. A proportional allocation method was then used, followed by systematic sampling, to visit households with eligible women. To determine the sampling interval (*K*), the total number of women of childbearing age in the selected kebeles (7045) was divided by the sample size (403), yielding *K* = 17. The first participant was selected using simple random sampling, and every 17th woman of childbearing age was selected to form the sample. In cases where two or more eligible women were present in the same household, a lottery method was used to select one participant.

### 2.5. Data Collection Tools

The data collection tool was adapted from previous relevant literature [11, 15–17], and the data were collected using structured and pretested interviewer administered questionnaires which were initially prepared in English and then translated into Somali language then back into English to check for any inconsistencies or distortion. The questionnaire contains sociodemographic related factors, obstetric factors, and health service related factors.

### 2.6. Data Collectors and Procedures

For data collection, five diploma nurses were recruited as data collectors, and two BSc‐degree nurses were appointed as supervisors. Mothers who were not available on the first day of data collection were revisited on subsequent days until the final day of the study. To ensure privacy, interviews with mothers were conducted in a separate, confidential space.

### 2.7. Variables of the Study

The study′s explanatory variables include age of mothers, residence, educational status, marital status, occupational status, religion, and family size, parity, nature of last pregnancy, ANC visit during last pregnancy, number of ANC visits in the last pregnancy, place of delivery, mode of delivery, any complication faced during delivery, advised about PNC during ANC visit, awareness of danger sign after giving birth, awareness of PNC, child sick during postnatal period, maternal care decision autonomy, and time to reach health facility.

### 2.8. Operational Definitions

#### 2.8.1. PNC Utilization

Mother who attends at least one PNC service or check up by health professional from health institution or health extension worker during the first 6 weeks starting immediately after the time of delivery and at any set up within the first 6 weeks of delivery (yes = 1, no = 0) [18].

#### 2.8.2. Awareness of Postpartum Danger Sign

If the mother mentions at least one postpartum complication of mother and newborn occur after birth such as vaginal bleeding, fever, edema, breast disease, unable to suck, vomiting everything and so on, it is coded as 1 (yes), and if not, it is coded as 0 (no) [18].

#### 2.8.3. Awareness of PNC

If the mother mentions at least one service from PNC services (health care education on danger signs that can occur during the postnatal period, counseling on breastfeeding and nutrition, childcare, immunization, family planning), it is coded as 1, and if not, it is coded as 0 (yes = 1,no = 0) [19].

#### 2.8.4. Maternal Autonomy Decision‐Making Power

It is the ability of a mother to exercise her autonomy in making choices related to her own health and well‐being, as well as that of her newborn child or children. This includes decisions about prenatal care, medical interventions during childbirth, infant feeding practices, and other important decisions that directly affect the mother and her child. In this study, if a mother decided by herself about the utilization of PNC services, then she is categorized as autonomous (1); otherwise, they were categorized as not autonomous (0) [20].

### 2.9. Data Quality Control

Data quality was ensured throughout the entire process, including during data collection, coding, entry, cleaning, and analysis. Two days of training were provided to data collectors and supervisors, covering the study′s relevance, objectives, ethical considerations, informed consent procedures, and interviewing techniques. The questionnaire was translated into the local language, Af‐Somali, and a pretest was conducted with 5% of the participants 2 weeks before the main data collection. This pretest was carried out in Garawo Kebele, which was not part of the study, to address any ambiguities in the questionnaire. Supervisors checked the completeness of the data daily and provided feedback to the data collectors the following morning.

### 2.10. Data Processing and Analysis

The data were entered using EpiData 4.6 and analyzed with SPSS Version 26. Descriptive statistics were used to summarize the characteristics of the participants, and the results were presented in text, tables, and figures. A binary logistic regression model was employed to assess the relationship between each independent variable and the outcome variable. In the bivariate analysis, independent variables with a *p* value of less than 0.25 were selected for further multivariate analysis. Multicollinearity was assessed using the variance inflation factor and standard error. The model′s fit was evaluated with the Hosmer–Lemeshow goodness of fit test. Adjusted odds ratios (AOR) with 95% confidence intervals (CIs) were reported, and a *p* value of less than 0.05 was considered statistically significant for PNC utilization.

## 3. Results

### 3.1. Sociodemographic Characteristics of Study Participants

A total of 403 mothers were included in this study with 100% response rate. The mean age of the mothers was 28.9 years (**S** **D** ± 5.8) and more than two‐third (60.3%) of mothers were in the age range of 25–34 years. Above three‐fourths 340 (84.4%), and 385 (95.5%) of the mothers were rural dwellers, and Muslim followers, respectively. Above three‐fourth, 368 (91.3%) and 303 (75.2%) of the mothers were married and housewives, respectively. Concerning the educational status of the mothers, the majority 283 (70.2%) had no formal education. More than two‐third, 250 (62.0%) of the mothers had more than five family members (Table 1).

**Table 1:** Sociodemographic characteristics of study participants in Degahbour District, Somali Region, Ethiopia (2023) (*n* = 403).

Variables	Category	Frequency	Percent
Residence	Urban	63	15.6
	Rural	340	84.4

Age of mother (in years)	≤ 24 years	86	21.3
	25–34years	243	60.3
	≥ 35years	74	18.4

Religion	Muslim	385	95.5
	Others ^∗^	18	4.5

Marital status of mothers	Married	368	91.3
	Divorced and widowed	35	8.7

Educational status of mothers	No formal education	283	70.2
	Primary (1–8)	74	18.4
	Secondary and above	46	11.4

Occupation status of mothers	Housewife	303	75.2
	Merchant	42	10.5
	Employed	17	4.2
	Daily laborer	41	10.2

Total family size	≤ 5	153	38.0
	> 5	250	62.0

^∗^Orthodox, Protestant, and Catholic.

### 3.2. Obstetric and Health Service–Related Characteristics of the Study Participants

Around 306 (75.9%) of mothers were multipara, and the majority of them 317 (78.7%) had planned pregnancy. Concerning the ANC of mothers, more than half 235 (58.3%) of mothers had visits, and nearly one‐third 114 (28.3%) of them visited ≥ 3 times. Regarding place of child birth, two‐thirds 242 (60%) of the mothers gave their last birth in the health institutions, and most of them 355 (88.1%) had delivered through spontaneous vaginal delivery. About obstetric complication faced during/after delivery of the last pregnancy, 115 (28.5%) of the mothers experienced at least one complication, whereas the rest were not. More than half 214 (53.1%) of the mothers reported that they are not autonomous to receive maternal health care, and 90 (22.3%) of the mothers reported that at least one episode of illness occurred in their child during postnatal period. More than half 203 (50.4%) of mothers, the average time to reach a health facility were 15–30 min. Nealy two‐third 232 (57.6%) of the mothers were advised by healthcare workers at health institutions during ANC visits. Regarding danger sign after give birth, more than half 229 (56.8%) of mothers had awareness about danger sign after give birth. Moreover, more than half 220 (54.6%) of mothers had not aware about the existence of PNC services after delivery (Table 2).

**Table 2:** Obstetric and health service related characteristics of the study participants in Degahbour District, Somali Region, Ethiopia (2023) (*n* = 403).

Variables	Category	Frequency (*n*)	Percent (%)
Parity	Primipara	97	24.1
	Multipara	306	75.9
Nature of last pregnancy	Planned	317	78.7
	Unplanned	86	21.3
ANC visit in the last pregnancy	Yes	235	58.3
	No	168	41.7
Number of ANC visit in the last pregnancy (*n* = 235)	1–2 visits	121	30.0
	≥ 3visits	114	28.3
Place of delivery	Home	161	40
	Health institution	242	60
Mode of delivery	Spontaneous vaginal	355	88.1
	Operative delivery	48	11.9
Any complication faced during delivery	Yes	115	28.5
	No	288	71.5
Maternal care decision autonomy	Autonomous	189	46.9
	Not autonomous	214	53.1
Child sick during postnatal period	Yes	90	22.3
	No	313	77.7

Time to reach health facility	< 15 min	109	27.0
	15–30 min	203	50.4
	> 30 min	91	22.6

Advised about PNC during ANC visits	Yes	232	57.6
	No	171	42.4
Awareness of danger sign after give birth	Yes	229	56.8
	No	174	43.2
Awareness of postnatal care	Yes	183	45.4
	No	220	54.6

### 3.3. Magnitude of PNC Utilization

**Figure 2:**
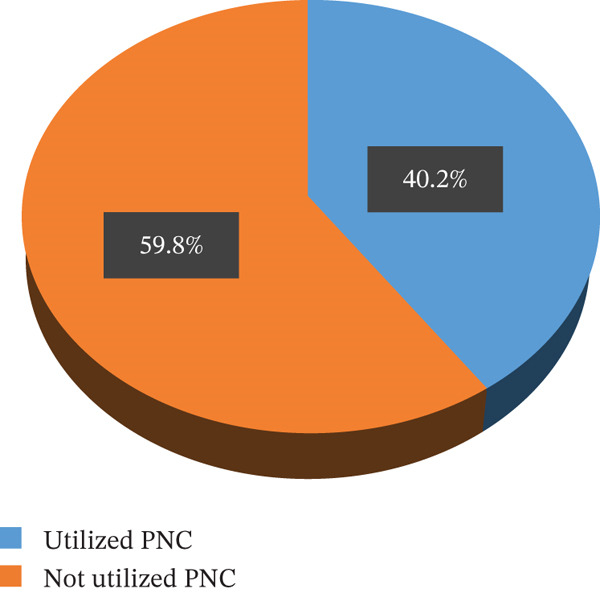
Magnitude of postnatal care service utilization among mothers in Degahbour District, Somali Region, Ethiopia (2023).

The magnitude of PNC service utilization among the mothers in Degahbour District, Somali Region was 40.2% (95% CI: 35.0, 44.9), that means 162 mothers use at least one PNC service, whereas the rest 241 mothers do not utilize PNC during the period of first 6 weeks (Figure 2).

### 3.4. Factors Associated With PNC Service Utilization

After controlling for possible confounding factors in the multivariable logistic regression analysis, educational status of mothers, ANC visit in the last pregnancy, place of delivery, complication faced during delivery, maternal care decision autonomy, and awareness of PNC were significantly associated with PNC utilization at a *p* value < 0.05. The odds of utilizing PNC were three times (AOR = 3.45; 95% CI: [1.61, 7.37]) higher among those mothers who had secondary and above education levels as compared with mothers who had no formal education. Mothers who were followed ANC during their pregnancy were three times higher (AOR 3.36; 95% CI [2.04, 5.54]) to utilize PNC compared with those who did not follow ANC during their pregnancy. The odds of utilizing PNC among mothers who delivered in health facilities were increased by 97% (AOR = 1.97; 95% CI: [1.20, 3.24]) as compared with those mothers who delivered at home.

Those mothers who faced birth related complication during delivery while giving birth was almost four times (AOR 3.93, 95% CI [2.33, 6.64]) more likely to get PNC services utilization than mothers who did not face complication while giving birth. The odds of PNC services utilization were 2.23 higher (AOR 2.23, 95% CI [1.39, 3.57]) among mothers who had autonomous to receive maternal care service comparing with mothers who were not autonomous. The odds of utilizing PNC among mothers who had awareness about PNC services were increased by 83% (**A** **O** **R** = 1.83; 95% CI: [1.14, 2.92]) as compared with whose mothers who had no awareness about PNC service (Table 3).

**Table 3:** Factors associated with postnatal care service utilization among women who gave birth in the last 6 months in Degahbour District, Somali Region, Ethiopia (2023) (*n* = 403).

Variables	PNCU		COR (95% CI)	AOR (95% CI)	*p*value
	Yes (%)	No (%)			
**Educational status of mother**					
No formal education	102 (36.0)	181 (64.0)	1	1	
Primary (1–8)	31 (41.9)	43 (58.1)	1.28 (0.76, 2.16)	1.17 (0.63, 2.15)	0.625
Secondary and above	29 (63.0)	17 (37.0)	3.03 (1.59, 5.78)	3.45 (1.61, 7.37) ^∗^	0.001
**Status of last pregnancy**					
Planned	139 (43.8)	178 (56.2)	2.14 (1.26, 3.62)	1.72 (0.93, 3.18)	0.084
Unplanned	23 (26.7)	63 (73.3)	1	1	
**ANC visit in the last pregnancy**					
Yes	120 (51.1)	115 (48.9)	3.13 (1.03, 4.83)	3.36 (2.04, 5.54) ^∗^	≤ 0.001
No	42 (25.0)	126 (75.0)	1	1	
**Place of delivery**					
Home	47 (29.2)	114 (70.8)	1	1	
Health institution	115 (47.5)	127 (52.5)	2.20 (1.44, 3.35)	1.97 (1.20, 3.24) ^∗^	0.007
**Complication faced during delivery**					
Yes	70 (60.9)	45 (39.1)	3.31 (1.12, 5.19)	3.93 (2.33, 6.64) ^∗^	≤ 0.001
No	92 (31.9)	196 (68.1)	1	1	
**Maternal healthcare decision autonomy**					
Autonomous	101 (53.4)	88 (46.6)	2.88 (1.91, 4.35)	2.23 (1.39, 3.57) ^∗^	0.001
Not autonomous	61 (28.5)	153 (71.5)	1	1	
**Time to reach health facility**					
≤ 30 min	140 (44.9)	172 (55.1)	2.55 (1.50, 4.33)	1.71 (0.92, 3.20)	0.092
> 30 min	22 (24.2)	69 (75.8)	1	1	
**Awareness on postnatal care**					
Yes	90 (49.2)	93 (50.8)	1.99 (1.33, 2.98)	1.83 (1.14, 2.92) ^∗^	0.012
No	72 (32.7)	148 (67.3)	1	1	

*Note:* The asterisk ( ^∗^) shows significance. 1.00: reference category.

Abbreviations: AOR, adjusted odds ratio; CI, confidence interval; COR, crude odds ratio; PNCU, postnatal care services utilization.

## 4. Discussion

This study is aimed at determining the magnitude of PNC service utilization and the factors associated with it among mothers who gave birth in the last 6 months in Degahbour District, Somali Region, Ethiopia. The findings revealed that the utilization of PNC services among these mothers was 40.2%. The study identified several factors significantly associated with PNC utilization, including the mother′s educational status, attendance at ANC during the last pregnancy, place of delivery, complications faced during delivery, maternal decision‐making autonomy regarding healthcare, and awareness of PNC services.

In this study, the magnitude of PNC service utilization among the mothers in Degahbour District, Somali Region was 40.2%. This result is consistent with the study conducted in Afghanistan, 44% [21], India 35.8% [22], Nepal 43.2% [23], Gondar 36.4% [24], Dessie 37% [25], and Gode 39.9% [15]. However, this finding is also higher than the studies conducted in Morocco 30.1% [26], and Nigeria 16.9% [27]. The difference might be due to differences in geographical barriers and the difference in study settings, where it was institutional‐based, and mothers may represent a fearful way of expressing their interest at the health institution in front of care providers, whereas it was a community‐based study in our study.

In addition, this finding is lower than the studies done in Malawi 84.8% [28], Sierra Leone 85.6% [29], and Ethiopia such as Damboya District 51.9% [18], Aysaeta District, Afar Region 45.1% [11], Loma District, South West Ethiopia 51.4% [30], Halaba Kulito Town 47.9% [19], and Sodo Zuria District 77.7% [31]. This discrepancy may be because mothers in this study area are less educated and less aware of the importance of PNC service utilization, and it might also be due to differences in study setting. In this study, above three‐fourths of study participants were rural residents, whereas in other studies the study participants were urban residents. Thus, mothers who reside in urban areas may have good awareness about the advantages of PNC service and have better educational status than rural residents. And mothers in the town may get easy access to health institution and health care providers when compared with rural residents.

In this study, mothers with secondary education or higher were three times more likely to utilize PNC compared with mothers who had no formal education. This finding is consistent with findings from Afghanistan [21], Nigeria [27], and Ethiopia such as South Gondar Zone [24], Dessie [25], Assela Town [32], and Fogera Woreda [33]. This could be because education plays an important role in enhancing female autonomy and assisting women in developing greater confidence and capability in making health‐related decisions for themselves. In addition, more educated mothers may have better knowledge and ideas about the benefits of PNC services to the health of mothers and their child [24].

In addition, mothers who attended ANC during their pregnancy were three times more likely to utilize PNC compared with mothers who did not attend ANC. This finding is in line with studies done in Afghanistan [21], Nigeria [27], Uganda [34], and Ethiopia such as Assela Town [32], Dolo Addo District [20], Gindeberet District [35], and Benchi‐Maji Zone [36]. This might be because information provided during ANC improves the knowledge of mothers′ better outcome of having postnatal care service utilization.

Furthermore, mothers who delivered in health facilities were 97% more likely to utilize PNC compared with those who delivered at home. This finding is in agreement with those of studies done in Afghanistan [21], Zambia [37], and Ethiopia such as Dolo Addo District [20], Halaba Kulito Town [19], Loma District, South West Ethiopia [30], Dessie [25], Debre Birhan Town [38], Debre Markos [39], and Sodo Zuria District [31]. This is because health education and appointment given for women after delivery might enhance the decision of women to utilize PNC during the postnatal period.

Mothers who experienced birth‐related complications during delivery were nearly four times more likely to utilize PNC services compared with those who did not face complications during childbirth. This finding is similar to the study done in Adigrat [40], Debre Markos [39], and Fogera Woreda, Ethiopia [33]. The possible explanation could be due to the fact that awareness of maternal complications is an important factor in motivating women and their families to attend health care services at the earliest opportunity with the intention of prevention and early detection, to be managed if any sign of complication occurs, have better decision for health, and have good attitude to go to health facility [39].

The odds of PNC services utilization were two times higher among mothers who had autonomy to receive maternal care service compared with mothers who were not autonomous. This is consistent with studies done in Dolo Addo District [20], Addis Ababa [41], and Sodo Zuria District [31]. This may be due to the fact that women′s equality often influences women to participate in all aspects including utilizing health care services, education, and politics.

The odds of utilizing PNC among mothers who had awareness about PNC services were increased by 83% as compared with those mothers who had no awareness about PNC service. This finding is consistent with studies done in Adigrat Town [40], Mekelle City [42], Tigray, Northern Ethiopia [40], Benchi‐Maji Zone; Southwest Ethiopia [36], Debre Birhan Town [38], and Fogera Woreda, Ethiopia [33]. This might be due to the reason that awareness can increase the demand for PNC services. Accordingly, it is important to provide health education to postpartum mothers before health facility discharge for creating awareness so as to enhance and sustain the use of PNC services.

The limitation of this study might be subject to recall bias since it was based on past events. In addition, since the study design is cross‐sectional, it is difficult to determine causal relationships between the proposed predictors and the outcomes of interest.

## 5. Conclusions

The utilization of PNC services in this study was found to be low compared with the Ethiopian Government′s Health Sector Transformation Plan and the national target for PNC coverage by 2020. Factors significantly associated with PNC utilization included maternal educational status, attendance of ANC during the last pregnancy, place of delivery, complications during delivery, maternal decision‐making autonomy, and awareness of PNC services.

### 5.1. Policy Implications

The findings of this study show the need for strengthened maternal health interventions aimed at improving PNC utilization. Efforts should focus on enhancing maternal awareness of the benefits of PNC through community‐based health education and counseling during antenatal and delivery services. Healthcare providers should strengthen counseling on PNC attendance, danger signs following childbirth, and the importance of institutional delivery. In addition, policies and programs that empower women in healthcare decision‐making and improve access to maternal health services at the community level may contribute to increased utilization of PNC services.

**Nomenclature**
ANC: antenatal care
AOR: adjusted odds ratio
CI: confidence interval
CSA: Central Statistical Agency
DDAAR: Degahbour District Administration Annual Report
EPNC: early postnatal care
FMOH: Federal Ministry of Health
IHRERC: Institutional Health Research Ethics Review Committee
SPSS: Statistical Package for Social Science

## Author Contributions

A.A.L. and H.M. were responsible for conceiving the research problem, initiating the study, writing the research proposal, and conducting the research. M.D. and A.A. handled data entry and analysis, wrote the interpretation of the results, and reviewed the final manuscript. Finally, all authors contributed to reviewing the manuscript.

## Funding

No funding was received for this manuscript.

## Ethics Statement

Ethical clearance was obtained from Institutional Health Research Ethics Review Committee (IHRERC) of Haramaya University College of Health and Medical Science (Reference Number IHRERC/043/2023) prior to conduct the study. Official permission letters, written by CHMS, were submitted to the Degahbour District administration. Informed, voluntary, written, and signed consent was obtained from the Degahbour District administration and each study participant. Additionally, informed, voluntary, written, and signed consent for those aged < 18 years was obtained from their guardians. To ensure confidentiality, any identifying information about the study participants was not indicated on the questionnaires, and they were informed that the collected data was used only for research purposes.

## Consent

The authors have nothing to report.

## Conflicts of Interest

The authors declare no conflicts of interest.

## Data Availability

The data sets used and/or analyzed during the current study are available from the corresponding author on reasonable request.
